# Predicting the effect of landscape structure on epidemic invasion using an analytical estimate for infection rate

**DOI:** 10.1098/rsos.240763

**Published:** 2025-01-08

**Authors:** Yevhen F. Suprunenko, Stephen J. Cornell, Christopher A. Gilligan

**Affiliations:** ^1^ Department of Plant Sciences, University of Cambridge, Downing Street, Cambridge CB2 3EA, UK; ^2^ Institute of Infection, Veterinary, and Ecological Sciences, University of Liverpool, Liverpool L69 7ZB, UK

**Keywords:** infection rate, analytical approximation, spatially explicit individual-based model, epidemiological model, epidemic invasion, crop landscape

## Abstract

The influence of landscape structure on epidemic invasion of agricultural crops is often underestimated in the construction and analysis of epidemiological models. Computer simulations of individual-based models (IBMs) are widely used to characterize disease spread under different management scenarios but can be slow in exploring large numbers of different landscape configurations. Here, we address the problem of finding an analytical measure of the impact of the spatial structure of a crop landscape on the invasion and spread of plant pathogens. We explore the potential of using an analytical approximation for the rate, r, at which susceptible crop fields become infected at the start of an epidemic to predict the effect that the spatial structure of a host landscape will have on an epidemic. We demonstrate the validity of this approach using two models: (i) a general IBM of the invasion and spread of a pathogen through an abstract host landscape; and (ii) an IBM of a real-life example for a virus disease spreading through a cassava landscape. Finally, we demonstrate that the analytical approach based on an estimate of the rate, r, can be used to identify spatial structures that effect deceleration of an invading pathogen.

## 1. Introduction

In recent years, considerable progress has been made in studying spatial dynamics to address broad-reaching challenges in biodiversity loss, food security and epidemiology [1–8]. In this study, we focus on the concept of spatial dynamics in epidemiology, more specifically we address invasion and spread of pathogens among plant populations in agricultural landscapes. Emerging epidemics of crop diseases continue to threaten food security in some of the world’s poorest regions [9–12]. Understanding the spatial aspects of invasion and spread of pathogens is an essential step in informing and optimizing the effective deployment of control measures, such as resistant varieties [13] and pesticides [14] to prevent and manage disease outbreaks in agricultural landscapes. A corollary of this is to consider how modifying the spatial configuration of agricultural crops in a landscape could decelerate the spread of pests and diseases [15,16].

The impact of spatial configurations of landscapes on ecological and epidemiological invasions has been studied using various approaches. These include deterministic partial differential and integro-differential equations [16–19], spatial metapopulation models [20], lattice and network models [21,22], interacting particle systems and spatial point processes [23]. Some of the most realistic and reliable estimations of the impact of spatial configurations of host landscapes on the invasion of plant pathogens in agricultural systems are frequently based on computer simulations of stochastic, spatially explicit, individual-based models (IBMs) of epidemics [7,8,24]. This can be sometimes excessively slow when multiple replicates are required for a range of parameter values in a spatially explicit IBM of pathogens spreading through highly resolved, realistic landscapes. Analytical approximations of IBMs can, in principle, be extremely efficient for quick identification of factors including pathogen, host and vector characteristics that could have a critical impact on disease spread.

To date, in life sciences, there has been limited success in formulating reliable analytical approximations for stochastic spatially explicit IBMs. For example, analytical approximations in the form of ‘moment closure’ [25,26] capture the important aspects of spatial structure but are non-rigorous and do not always guarantee to give the correct answer [23,27]. Ovaskainen *et al*. [28,29] developed a more reliable approximation scheme for IBMs that provided asymptotically exact results when interactions between individuals were sufficiently long-ranged. Further advances by Cornell *et al.* [30] broadened the scope of the approximation scheme proposed by Ovaskainen *et al.* [28,29] to a wider range of IBMs in life sciences. In spatial ecology and epidemiology, the approximation scheme developed in [28,29] has been used by several authors [20,31] to derive the invasion threshold, $R_{0}=1$, for epidemics, where $R_{0}$ is the basic reproduction number. North & Godfray [20] derived the invasion threshold for spatially explicit metapopulations in dynamical landscapes; Suprunenko *et al.* [31] derived a threshold for localized invasion in IMBs with randomly distributed hosts. In the case of randomly distributed hosts in IBMs, Wadkin *et al.* [32] introduced an expression for $R_{0}$ taking into account host depletion, while van den Bosch *et al.* [33] using the next-generation matrix approach and the *O*-ring statistic introduced an expression for $R_{0}$ applicable for non-random host distributions and for a multiple host case.

The goal of this study is to investigate the reliability of analytical approximations of epidemiological IBMs in assessing the impact of the spatial distribution of hosts across a landscape on pathogen invasion. Specifically, we address the following questions: (i) Can the impact of landscape reconfiguration on epidemic invasion and spread be predicted analytically? and (ii) Can an analytical approach be useful for selecting from a set of spatial configurations those that decelerate the dispersal of an invading pathogen?

To answer these questions, we consider the possibility of using an analytical expression for the rate, r, at which a population of susceptible fields become infected at the start of an epidemic as a quantitative measure of the impact of landscape structure on the invasion and spread of crop pathogens. The product of the infection rate, r, and the mean infectious period, τ, provides an approximation for the basic reproduction number, $R_{0}\approx r\times \tau$, and can be used to study the invasion threshold, i.e. $R_{0}=1$. To derive an analytical approximation for the infection rate, we use the mathematical framework for the analysis of IBMs in life sciences introduced by Ovaskainen *et al.* [28,29] and developed by Cornell *et al.* [30]. Within this framework [28–30], we analyse two IBMs to address specific research questions. The first IBM, Model 1, is a general model of the invasion and spread of an abstract pathogen through an abstract landscape of immobile susceptible hosts where we consider different spatial reconfigurations of the host landscape. Within this framework [28–30], we derive an analytical approximation for the infection rate r for Model 1. To test the reliability of the analytical approximation for r for each landscape configuration we compare the predicted infection rate with rates derived from computer simulations of epidemic invasion in Model 1. By considering different spatial configurations and their corresponding analytical predictions for r, we test the use of r to select from different landscape configurations those that decelerate the dispersal of an invading pathogen.

In the second IBM, referred to as Model 2, we consider the invasion and spread of the cassava brown streak virus (CBSV) through a cassava landscape typical of sub-Saharan Africa. The goal of considering Model 2 is to test the applicability of the analytical approach to real-life diseases and heterogeneous host landscapes that differ from the simplified formulation in Model 1. Datasets that describe real-life host landscapes are often incomplete and lack detailed information about host spatial locations. In that case, usually, a host landscape is rasterized as a grid of cells where each cell is characterized by the number of hosts within that cell, but no exact locations of those hosts are provided. We use the analytical approximation for the infection rate derived in this article and adapt it for the invasion and spread of a pathogen through a rasterized heterogeneous host landscape. Also, in real-life systems the parameters of pathogen dispersal are seldom known exactly and instead are estimated by fitting the model to the surveillance data. This results in a probability distribution for dispersal parameters, which we sample to assess the effects of uncertainty on the performance of the analytical approximation in predicting epidemic behaviour, when compared with computer simulations. Finally, we test the ability of the analytical approach to identify real-life host landscape reconfigurations that could decelerate invading pathogens.

## 2. Method

### 2.1. Individual-based model 1: invasion and spread of an abstract pathogen over different configurations of a host landscape

**Figure 1.**
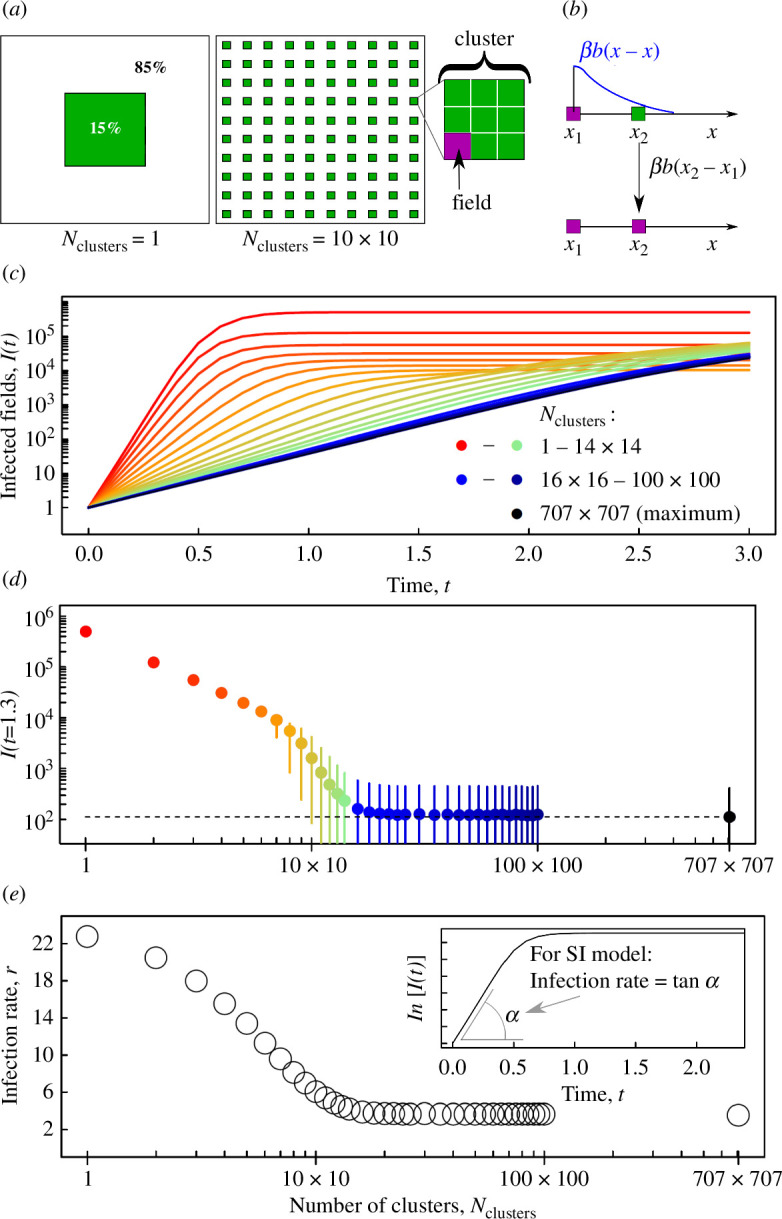
Effect of landscape configuration on epidemic progress in an agricultural crop landscape. (*a*) Two examples of different spatial configurations described by the number of identical clusters $N_{clusters}$ of the crop area (green) consisting of identical square fields (a field is an individual host unit in the IBM 1). The total crop area in all cases here represents 15% of the total area of a landscape. (*b*) Within the IBM 1, the probability that a susceptible individual field (green) is infected from an infected field (magenta) depends upon the product of a transmission rate and a dispersal kernel, $\beta b\left(x\right)$, for the pathogen. (*c*) Dynamics of the expected number of infected fields I(t) obtained from computer simulations of the IBM 1. (*d*) Values of I(t), shown as points, at an arbitrarily selected time, t=1.3 after invasion, in the interval between t=0.5 and t=1.5 where trajectories are well separated and can be easily distinguished visually; the intervals between 2.5% and 97.5% percentiles (shown as vertical bars) in the distribution of the number of infected fields in individual simulations. (*e*) Infection rate of susceptible fields becoming infected at the start of an epidemic estimated from simulations according to equation (2.1) using $I\left(0\right)=1$ and δt=0.1. Data and codes for all figures in this article are available from Figshare [34,35].

Model 1 is a compartmental epidemiological IBM of pathogen invasion and spread through a population of fields in which a host crop is growing in an agricultural landscape. Fields containing the host crop may be in one of two states, susceptible (S) or infected (I); other fields are uninfectable because they are sown to other crops. For simplicity and ease of presentation, we assume identical square fields, with area $A_{0}$, that occupy a total area $A_{H}$ in a square agricultural landscape, of area *A*, where $A_{H}<A$. An IBM treats an individual field as a discrete point-like host at the centre of that field. The epidemic model is focused on the landscape scale and the increase in disease within the fields is not modelled. Different spatial configurations of the host crop landscape are obtained by aggregating fields into clusters of different sizes. The clusters are arranged spatially on a regular square grid (cf. selected illustrations for 1 and 100 clusters in figure 1*a*) and the number of clusters is denoted as $N_{clusters}$. Fields within a cluster are also arranged on a regular square grid, without overlap or gaps between fields (figure 1*a*).

Infection of a susceptible field from an infected field a distance x away is described by two components: (i) the parameter β describing the infection rate per contact density; and (ii) a normalized dispersal kernel, $b\left(x\right)$, described here by a Gaussian function $b\left(x\right)=\;1/2\pi \sigma^{2}\exp\left(-x^{2}/2\sigma^{2}\right)$ with standard deviation σ (figure 1*b*). We assumed that the pathogen was introduced at time t=0 in a single randomly selected field. Epidemics were simulated using the ModelSimulator software presented by Cornell *et al.* [30]. The following parameters were used (figure 1*c*–e): the area of an individual field $A_{0}=0.04$ km^2^; the total number of hosts N=500,000; the area, $A_{H}$, occupied by an agricultural crop represents 15% of the total area, $A=A_{H}/0.15\approx 365\times 365$ km^2^; σ=10 km; time was measured in units where the rate β=1. The expected number of infected fields, I(t), was calculated as an average over 2000 simulations.

### 2.2. Infection rate and its analytical approximation

### 2.2.1. Infection rate

The rate at which susceptible fields become infected at the start of an epidemic is defined as the exponential growth rate of the expected number, I(t), of infected fields at sufficiently small time t=δt after $I\left(0\right)$ fields were infected at time t=0,


(2.1) $$I\left(\delta t\right)\approx I\left(0\right)e^{r\;\delta t}.$$

### 2.2.2. Analytical approximation for the infection rate

To derive an analytical approximation for the infection rate, we use the mathematical framework [28–30] for the analysis of general IBMs in life sciences. Within this framework [28–30] the analytical approximation for the time-dependent expected density $Q_{m}(t)$ of individuals of type m (e.g. infected or susceptible individuals per unit area) is represented by the following expansion [30]:


(2.2) $$Q_{m}\left(t\right)=q_{m}\left(t\right)+p_{m}\left(t\right)+o\left(\frac{1}{L^{2}}\right),$$

where L is the typical length scale of interactions between individuals, $q\left(t\right)$ is the leading contribution given by the mean-field density (it is identical to the density in a classical non-spatial model, $q_{m}\left(t\right)=\underset{L\to \infty }{\lim}Q_{m}(t)$); $p_{m}\left(t\right)$ is the leading correction, $p_{m}\left(t\right)∼1/L$; other corrections are of a higher order of the small parameter, 1/L. For applications in systems where L is sufficiently large, it is sufficient to consider only the leading contribution $q_{m}(t)$ and the first-order correction term $p_{m}(t)$ [29,30].

Here, in the case of an epidemiological IBM for the spread of disease through a landscape of immobile hosts (Model 1), we use the same approach, i.e. we use $Q_{m}\left(t\right)=q_{m}\left(t\right)+p_{m}\left(t\right)$, and derive the dynamical equations for the leading contributions $q_{S}(t)$ and $q_{I}(t)$ in Model 1:


(2.3) $$\frac{dq_{S}\left(t\right)}{dt}=-\frac{dq_{I}\left(t\right)}{dt}=-\beta q_{S}\left(t\right)q_{I}(t).$$

The dynamics of corrections $p_{S}(t)$ and $p_{I}(t)$ are determined by the dynamics of densities q(t) and spatial cumulants $g_{SI}(x,t)$ [29,30]:


(2.4) $$\frac{dp_{S}\left(t\right)}{dt}\;=-\frac{dp_{I}\left(t\right)}{dt}\;=-\;\beta \left[\int_{0}^{\infty }2\pi xb\left(x\right)g_{SI}\left(x,t\right)dx+\left(\;p_{I}\;q_{S}\;+\;p_{S}\;q_{I}\right)\right].$$

The following initial conditions are used: $q_{S}\left(0\right)=n_{S}$, $q_{I}\left(0\right)=n_{I}$ and $p_{S}(0)=p_{I}(0)=0$. Here, $n_{S}$ (or $n_{I}$) represents the spatial density of susceptible (or infected) fields within a landscape, the total density of fields is denoted as n, where $n=n_{S}+n_{I}$. Following the idea that the invasion is a localized phenomenon [31], we use $n_{S}=(N-I(0))/A$, $n_{I}=I(0)/A$, where A is the area where the number of fields, $I\left(0\right)$, were infected at time t=0, and where N is the total number of susceptible and infected fields within the area A at time t=0. Using the expression for the infection rate $r=Q_{I}^{-1}(t)dQ_{I}\left(t\right)/dt{\left.\right|}_{t\to 0}$, and substituting the initial condition and the expression for $dQ_{I}/dt{\left.\right|}_{t\to 0}$ derived from equations (2.2)–(2.4), the expression for the infection rate r becomes (also see [20,25,31,33]):


(2.5) $$r=\beta \times \left(n_{S}+\frac{1}{n_{I}}\int_{0}^{\sqrt{A}/2}2\pi xb\left(x\right)g_{SI}\left(x,t=0\right)dx\right),$$

where the spatial cumulant $g_{SI}\left(x\right)$ is calculated on the local square area A, therefore it takes values on the interval $x\in (0,\sqrt{A}/2)$, hence the integral is calculated on the same interval. The mathematical expression (2.5) applies to a broad class of epidemiological models including SI, SIS, SIR, SIRS and the SEI(R)(S) family, where R and E denote removed and exposed classes, respectively (see electronic supplementary material, supplementary notes S1 and S2).

To derive the approximation for $g_{SI}(x,t=0)$, we consider second-order spatial cumulants, $g_{SI}(x)$ and g(x), and second spatial moments, $k_{SI}^{\left(2\right)}(x)$ and $k^{\left(2\right)}(x)$. Here, $k_{SI}^{\left(2\right)}\left(x\right)$ is proportional to the probability of there being a susceptible and an infected host a distance x apart, and $k^{\left(2\right)}\left(x\right)$ is proportional to the probability of there being two hosts (of any type) at distance x from each other [29]. Spatial cumulants and moments are connected by the following relationships [29]: $k_{SI}^{\left(2\right)}\left(x\right)=g_{SI}\left(x\right)+n_{s}n_{I}$ and $k^{\left(2\right)}\left(x\right)=g\left(x\right)+n^{2}$. From the standard assumption [20,25] that the initial introduction of a pathogen in a population of N fields occurs by infecting a number I(0) of randomly selected fields, it follows that a randomly selected field is infected with probability $n_{I}/n$ and susceptible with probability $n_{S}/n$. Therefore, we get: $k_{SI}^{\left(2\right)}\left(x\right)=k^{\left(2\right)}\left(x\right)\frac{n_{I}n_{S}}{n^{2}}$ and, consequently,


(2.6) $$g_{SI}\left(x\right)=g\left(x\right)\frac{n_{I}n_{S}}{n^{2}}.$$

An alternative derivation of the relationship (2.6) using an auxiliary IBM and the mathematical framework introduced by Ovaskainen *et al.* [29] is presented in electronic supplementary material, supplementary note S3. For comparison of the approximation (2.6) with other existing approximations [20,25,33] that assume $g_{SI}\left(x\right)=g\left(x\right)n_{I}/n_{S}$ see electronic supplementary material, supplementary note S4. Substituting the approximation (2.6) into the expression (2.5), we obtain the following expression for the infection rate that is used in this work:


(2.7) $$r=\beta \times \left(n_{S}+\frac{n_{S}}{n^{2}}\int_{0}^{\sqrt{A}/2}2\pi xb\left(x\right)g\left(x\right)dx\right).$$

### 2.2.3. Testing the analytical estimate (2.7)

**Figure 2.**
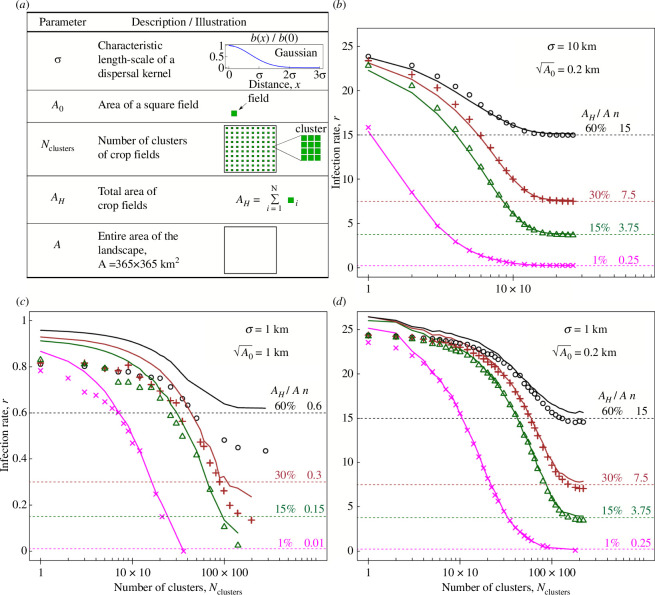
Performance of the analytical approximation for epidemic infection rate in IBM (equation 2.7), for different parameter values. (*a*) Parameters used in the IBM. The following values were considered: σ=1 km and 10 km; $\sqrt{A_{0}}=$0.2 km and 1 km; $N_{clusters}$ varying from 1 until approximately 200×200;$A_{H}$ from 1% to 60% of A, where A is fixed as 365×365 km^2^. (*b–d*) Analytical estimates (solid lines) were compared with estimates from computer simulations (symbols) for different spatial scales of pathogen dispersal σ and landscapes. The infection rate is estimated from results of computer simulations in the same way as in figure 1*e*. Note, according to equation (2.7), the maximal value of r at $N_{clusters}=1$ is close to the value $r_{max}=\beta n_{max}$, where $n_{max}\approx 1/A_{0}$, therefore $r_{max}=25$ in panels (*b*) and (*d*), and $r_{max}=1$ in panel (*c*). Results on the panel (*c*) for each value of $A_{H}/A$ separately are shown in electronic supplementary material, supplementary note S5.

Using computer simulations of Model 1, we consider different values of model parameters describing landscapes and pathogen dispersal (figure 2*a*). Three different relationships between σ, a characteristic length scale of dispersal and $\sqrt{A_{0}}$, a characteristic length scale of host landscape are considered: (i) $\sigma \gg \sqrt{A_{0}}$ (figure 2*b*); (ii) $\sigma >\sqrt{A_{0}}$ (figure 2*d*); and (iii) $\sigma =\sqrt{A_{0}}$ (figure 2*c*). The case corresponding to the relationship $\sigma <\sqrt{A_{0}}$ is not considered as relevant to epidemics in agricultural systems. Also, we consider different amounts of crop area, $A_{H}$, within a fixed landscape A, different levels of fragmentation of the spatial configuration parameterized by the number of clusters $N_{clusters}$, two values of the parameter σ and two values of $\sqrt{A_{0}}$. For estimates using equation (2.7), densities n and $n_{S}$, and cumulants $g\left(x\right)$ are calculated for the whole landscape A; cumulants are calculated using the function spm2 from ModelSimulator software provided in [30] using the size of a single field, $\sqrt{A_{0}}$, as a grid resolution (i.e. an input parameter for the function spm2). For estimates of r using smaller local areas in the case $\sigma =\sqrt{A_{0}}$ , see electronic supplementary material, supplementary note S6.

### 2.3. Individual-based model 2: invasion and spread of CBSV over different configurations of cassava landscapes

We consider the analytical approach tested for Model 1 and apply it to the invasion and spread of CBSV through an example of a cassava landscape in sub-Saharan Africa [36]. Cassava is used as an example of an important crop whose production is under threat from several pests and pathogens. Cassava is a root crop that is important for food security and economic stability in sub-Saharan Africa [37]. The ongoing spread of cassava brown streak disease was initially reported in Uganda in 2004 [38], and recently it has been reported in the northcentral part of the Democratic Republic of the Congo [39].

### 2.3.1. Cassava landscape and its spatial reconfigurations

**Figure 3.**
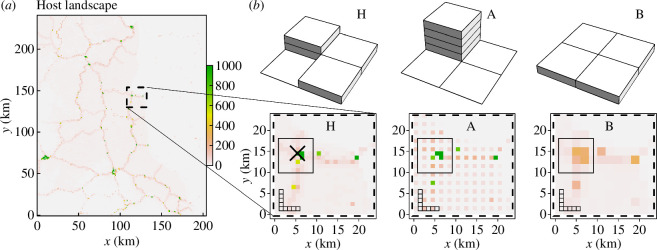
A default (original) cassava landscape and two reconfigurations in Model 2. (*a*) A sample area of the original rasterized cassava landscape map provided by Szyniszewska *et al.* [36] and converted to fields [24] (§2). (*b*) A sample area of the original landscape, denoted H, and its spatial re-configurations, denoted A and B; all three configurations are at 1 km resolution. Dashed and solid rectangles show 24 km-by-24 km and 8 km-by-8 km areas, respectively, that are used to estimate the infection rate analytically. The primary infected field is located at the intersection of solid black lines shown in landscape H in panel (*b*).

To date, the most detailed available map for cassava landscapes is provided in [36], where the data on cassava harvested area in sub-Saharan Africa are resolved in a raster at 1 km resolution, i.e. the data provide estimates of the total area of cassava fields located within each 1 km-by-1 km raster cell. However, the exact locations and geometry of individual fields are not specified in any given cell. A sample of the cassava landscape in sub-Saharan Africa extracted from [36] is shown in figure 3*a*.

To test the impact of spatial configuration of fields on epidemic dynamics, we consider the cassava landscape on a square lattice with mesh size equal to 2 km where each 2 km-by-2 km cell contains four original 1 km-by-1 km raster cells. We assume that reconfigurations can move cassava fields within 2 km-by-2 km cells, but the total area of cassava fields within each 2 km-by-2 km cell remains constant. From this class of reconfigurations, we consider two contrasting types, denoted as type A and type B, representing opposite extremes of a spectrum: type A denotes aggregation of fields within 1 km-by-1 km cells for each 2 km lattice; type B denotes homogenization of fields across 1 km-by-1 km cells for each 2 km lattice. These reconfigurations are constructed as follows:

— *Type A reconfiguration* (aggregation within 2 km lattice)—the total area of cassava fields in each 2 km-by-2 km cell is placed onto a single 1 km-by-1 km original raster cell until its maximal capacity is reached, the excess is placed onto the next 1 km raster cells until its maximal capacity is reached and so on. Cells are filled in the following sequence: the North-Western cell is first, then North-Eastern, South-Western and finally South-Eastern cells. All fully occupied cells in the original unmodified landscape were assumed to remain fully occupied.
— *Type B reconfiguration* (homogenization within 2 km lattice)—the total area of cassava fields in each 2 km-by-2 km cell is divided equally among four original 1 km-by-1 km raster cells.

Thus, we consider three landscapes: (i) the original landscape denoted as H; (ii) the landscape A; and (iii) the landscape B. These three landscapes are illustrated in figure 3*b*, also see table 1.

**Table 1.** Notation used in connection with Model 2.

Symbols	Description
*t*	time. units: years
H,A,B	landscapes: H is original, A and B are modified by reconfiguring H as described in §2
b(x)	pathogen dispersal kernel
β,α,p	parameters of dispersal: β is the infection rate per contact density, p is the proportion of dispersed inoculum that remains in the source cell and α is the kernel exponent
$I\left(t\right)$	number of infected fields at time t
$I_{j}\left(1/12\right)$ $I_{j}\left(0.5\right)$ j=H,A,B	number of infected fields at time t=1/12 year or t=0.5 year in one of the three landscape configurations: H, A or B
r	rate of susceptible fields becoming infected at the start of an epidemic (equation 2.1). Units: year^–1^
$r_{j}$ 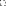 j=H,A,B	rate r estimated analytically in one of the three landscape configurations (H, A or B) on the area outlined by dashed contour
$r_{j}$ 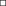 j=H,A,B	rate r estimated analytically in one of the three configurations of a landscape (H, A or B) on the area outlined by solid black contour
exp⁡(r/12)	the number of infected fields estimated at t=1/12 year assuming exponential growth, exp⁡(r×t)

### 2.3.2. Invasion and spread of CBSV through cassava landscapes

Model 2 represents a modified version of Model 1 with a realistic landscape that is derived from the cassava landscape in sub-Saharan Africa extracted from [36]. The pathogen’s dispersal characteristics are derived from the work of Godding *et al*. [24] who fitted a raster-based CBSV spread model to the data for CBSV spread in Uganda. Details of the derivation are specified below.

We use the cassava harvested area in an arbitrarily selected region on the border between Cameroon and Central African Republic extracted from CassavaMap from [36] (figure 3*a*). We apply the same discretization as presented by Godding *et al*. [24], i.e. the area of a single unit field of cassava is 0.1 ha, one raster cell can have a maximum of 1000 cassava fields; the actual number of hosts in each raster cell is rounded down to the nearest integer number. A sample of the discretized host landscape is shown in figure 3*a*. In a raster-based epidemic spread model [24], all hosts within a raster cell interact with each other with the same constant infection rate. To reproduce this feature, we place all hosts within each raster cell exactly at the centroid of that cell.

**Figure 4.**
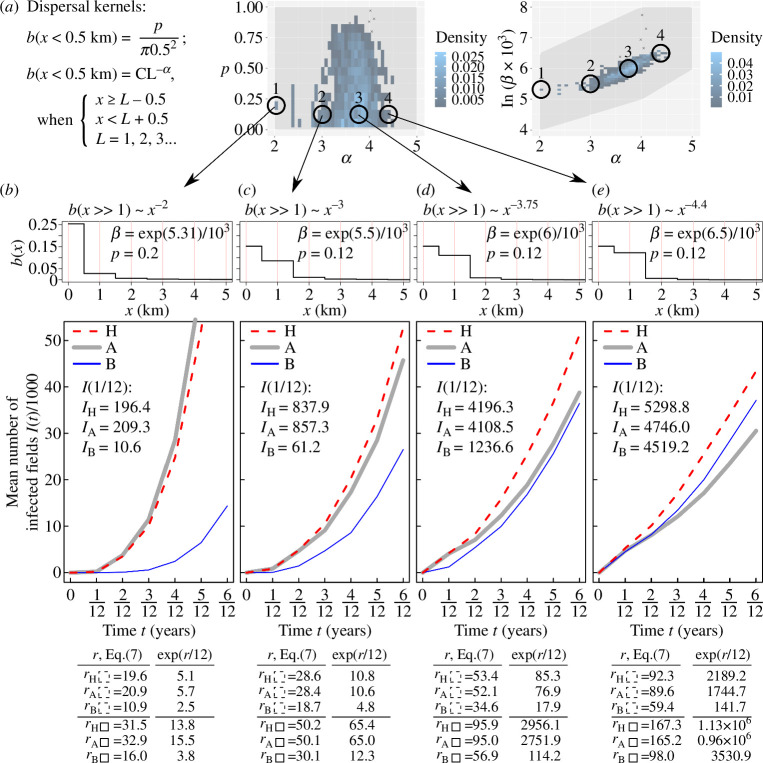
Computer simulations of an IBM of invasion and spread of CBSV through a default (original) cassava landscape and two reconfigurations (Model 2 defined in §2). (*a*) Parameters of pathogen dispersal kernels b(x) sampled from posterior distribution of parameters p, α and β (modified from figure 3 in Godding *et al.* [24]). (*b*–*e*) Each column shows the dispersal kernel, corresponding results of computer simulations, and the estimates of infection rate calculated from simulations, and from equation (2.7) using 24 km-by-24 km area (denoted by r with a dashed rectangle as a subscript) and 8 km-by-8 km area (denoted by r with a solid rectangle as a subscript); these areas are shown in figure 3*b*. Estimates of infection rate from equation 2.7) were used to calculate the number of infected fields, exp⁡(r×Δt), assuming exponential growth for Δt=1/12 year and compared with the mean value $I\left(\Delta t\right)$ estimated from simulations. The median, 5% and 95% percentiles for 1000 individual simulations related to $I\left(\Delta t\right)$ are shown in electronic supplementary material, figures S5 and S6, supplementary note S7.

In Godding *et al.* [24] the pathogen’s dispersal probability is described by a raster that is determined by the parameter β (the infection rate per contact density) and a discrete dispersal function $K\left(d_{ij}\right)$ determined by the distance $d_{ij}$ between centroids of the i-th and j-th raster cells: $K\left(d_{ij}=0\right)=p$ and $K\left(d_{ij}>0\right)=Cd_{ij}^{-\alpha }$, where the constant C is the normalization constant, p is the proportion of dispersed inoculum that remains in the source cell and α is the kernel exponent. Here, using function $K\left(d_{ij}\right)$, we derive a radially symmetric dispersal kernel: we consider the discrete values of $K\left(d_{ij}\right)$ taken along the direction of increasing longitude, and use those values to define the staircase-like radially symmetric function $b\left(x\right)$ (figure 4*a*). Aiming to test the applicability of the analytical approximation rather than to predict the cross-continental epidemic spread, we consider the epidemic spread only within a relatively small fraction of a cassava growing region (figure 3*a*) and truncate the function $b\left(x\right)$ at 50 km, b(x>50 km)=0. The resulting function $b\left(x\right)$ is used as a dispersal kernel in the Model 2.

**Figure 5.**
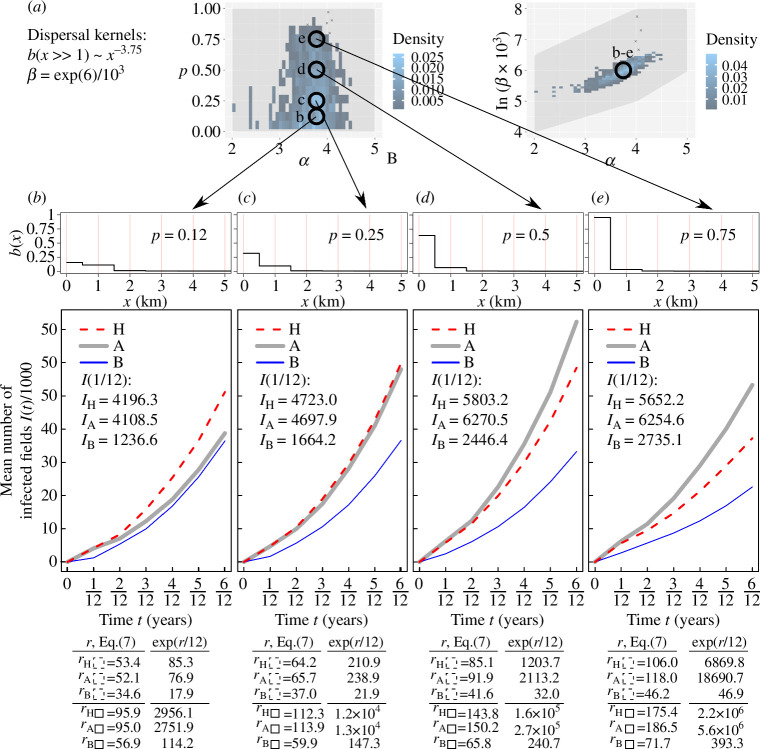
Computer simulations of an IBM of invasion and spread of CBSV through a default (original) cassava landscape and two reconfigurations (Model 2 defined in §2), part 2. Same as figure 4 but using dispersal kernels with fixed parameters α and β and different values of the parameter p defined in (*a*). (*a*) Parameters of pathogen dispersal kernels b(x). (*b–e*) Dispersal kernels, results of computer simulations and estimates of the infection rate calculated from simulations and equation (2.7). Note, the dispersal kernel in panel (*b*) corresponds to the dispersal kernel in figure 4*d*.

Using the results for parameter estimation provided by Godding *et al.* [24], we consider several sets of different parameter values for β,p and α: four sets are shown in figure 4*a*; the other four sets with fixed values for α and β are shown in figure 5*a*. Note that in this study, β is measured in units [number of fields per area]^–1^ year^–1^, while in Godding *et al.* [24] it is measured in units [one raster cell per area]^–1^× year^–1^, where one raster cell corresponds to 1000 individual fields. Thus, the value of β in Godding *et al.* [24] corresponds to a thousand times smaller value of β in this study. For example, the first set of parameter values shown in figure 4*a* is: $\beta \approx 0.001\times \exp\left(5.31\right)$$km^{2}{year}^{-1}$, $p=0.2km^{-2},$ α=2; values for all other sets of selected parameter values are shown in figures 4*a* and figure 5*a*.

For each set of model parameters, 1000 individual simulations were performed assuming a single initially infected field at the same location (marked by the intersection of two black lines, figure 3*b*); here, the location of primary infection is inside a maximally occupied cell (i.e. 1000 fields in a 1 km-by-1 km cell). A case when the primary infection is placed in the less populated area (40 fields in a 1 km-by-1 km cell) is considered in electronic supplementary material, supplementary note S7. To estimate the size of an epidemic at short term and long term after the start of an epidemic, we calculated the mean number of infected fields at one and six months, denoted by I(t=1/12year) and I(t=0.5year), respectively, shown as insets on panels in figures 4*b*–e and 5*b*–e, also see table 1; for median value as well as 5% and 95% percentiles, see electronic supplementary material, supplementary note S7. Note, in Model 2 we do not estimate infection rates at the start of an epidemic from computer simulations as was done in Model 1. Instead, we test the ability of the analytical approach to characterize the longer-term epidemic dynamics (i.e. the number of infected hosts one and six months after the start of an epidemic) and to identify from the selected specific host landscapes those that could reduce the number of infected hosts in the longer term.

### 2.4. Analytical estimation of the infection rate in Model 2

In heterogeneous landscapes, infection rates should be estimated locally in space because the invasion of a pathogen is a localized phenomenon [31]. To explore the role of the size of the local area, we estimate the infection rates in areas of two sizes: 24 km-by-24 km (denoted by dashed black contour, figure 3*a*) and 8 km-by-8 km (denoted by black solid contour, figure 3*b*), also see table 1. Note that all dispersal kernels b(x) considered here decay significantly for x>2 km, therefore both the selected areas for calculating *r* are larger than the characteristic dispersal scale in all kernels. For each selected area, we estimate parameters $n_{S}$ and n, and calculate the function $g\left(x\right)$ using ModelSimulator software provided by Cornell *et al.* [30]; we use 1 km (a size of a raster cell) as a grid resolution. For each of the selected dispersal kernels (figure 4*a*), the theoretical estimates equation (2.7) denoted as r are shown as insets in panels figures 4*b*–e and 5*b*–e. To compare analytical estimates with computer simulations, we estimate the number of infected fields at time Δt=1/12 years as exp⁡(r×Δt) assuming exponential growth with the estimated rate r; values of exp⁡(r×Δt)=exp⁡(r/12) are shown as insets in panels figure 4*b*–e.

## 3. Results

### 3.1. Infection rate

Computer simulations of Model 1 demonstrate that the infection rate can be used to infer the effect of the spatial structure of the host landscape on epidemic dynamics (figure 1). Redistributing crop fields among separated clusters ($N_{clusters}$), while retaining the total area of crop ($A_{H}$) in a domain (A) decelerated the invasion and spread of a pathogen (figures 1*c*–e and 2), unless the initial configuration was already fragmented into a large number of clusters.

### 3.2. Analytical estimate of infection rate

We derived the analytical expression (equation 2.7) for the infection rate r that applies to the compartmental SI epidemiological model as well as other models from the S(E)I(R)(S) family (electronic supplementary material, supplementary notes S1 and S2). There was good agreement between values of r estimated from simulations and analytically (figure 2*b*–d), when the pathogen’s dispersal was sufficiently long-ranged relative to field size. The agreement between the analytical and simulation-derived estimates was improved either by increasing the characteristic spatial scale of dispersal, σ (cf. transition from figure 2*d* to figure 2*b*), or by lowering the characteristic scale of spatial distribution of hosts, $\sqrt{A_{0}}$ (cf. transition from figure 2*c* to figure 2*d*). The case corresponding to the relationship $\sigma =\sqrt{A_{0}}$ (figure 2*c*) illustrated the limits of the applicability of the underlying analytical framework [30], with deviation between the analytical approximation and simulation increasing as the proportion of domain occupied by the susceptible crop increases.

### 3.3. The impact of spatial reconfigurations of cassava landscape on invasion and spread of CBSV

Considering Model 2 for the invasion and spread of CBSV through an example of a cassava landscape and its reconfigurations, we tested the applicability of the analytical approximation (equation 2.7) to real-life systems. Here, we first summarize results obtained from computer simulations, and then compare them with results obtained by analytical estimates using equation (2.7).

Computer simulations indicated that small-scale spatial reconfigurations of the host landscape (figure 3*b*) can have a substantial effect on the epidemic (figures 4*b*–e and 5*b*–e). In particular, using the size of an epidemic in landscape H as a baseline, type A reconfiguration (i.e. local aggregation) changed I(t=0.5) within the range from +43% to −30% (figures 5*e* and 4*e*, respectively). Type B reconfiguration (i.e. local homogenization) decreased the value for I(t=0.5), with the largest observed deviation of −84% (figure 4*b*). The effect of landscape reconfigurations on an epidemic under Model 2 has a complicated dependence on the relationship between the host landscape and the dispersal kernel of the pathogen. For example, when dispersal within a source cell is dominant, i.e. b(x=0)≫b(x>0) (cf. figure 4*b*), local aggregation of hosts (type A) results in a larger epidemic than for either of the other two landscapes, i.e. $I_{A}\left(t\right)>I_{H}\left(t\right)$, $I_{A}\left(t\right)>I_{B}\left(t\right)$ (figure 4*b*). However, when dispersal to nearest neighbours is comparable to the dispersal within a source cell, i.e. $b\left(x=0\right)\approx b\left(x=1\right)$ (cf. figure 4*d*), the effect of local aggregation (type A reconfiguration) depends on the landscape: for example, in figure 4*d* type A aggregation of hosts decelerates an epidemic when compared with landscape H, $I_{A}\left(t=0.5\right)<I_{H}\left(t=0.5\right)$, but accelerates an epidemic when compared with landscape B, $I_{A}\left(t=0.5\right)>I_{B}\left(t=0.5\right)$.

As the key result obtained from Model 2, we found that the analytical estimates of the infection rate correctly identified the relative ranking of the number of infected fields for landscapes H, A and B for each dispersal kernel at short term and medium term. Using I(t=1/12) as a metric for the short term and I(t=0.5) as a metric for the medium term, the relations such as, for example, $I_{A}\left(t\right)>I_{H}\left(t\right)>I_{B}\left(t\right)$ in figure 4*b* or $I_{H}\left(t\right)\approx I_{A}\left(t\right)>I_{B}\left(t\right)$ in figure 4*c* or $I_{H}\left(t\right)>I_{A}\left(t\right)>I_{B}\left(t\right)$ in figure 4*d* were correctly inferred from estimates of r. Thus, estimates of r determined if a spatial reconfiguration of the host landscape would, on average, increase I(t), decrease or make no practical difference. An exception occurred for the dispersal kernel in which dispersal within the cell and to the nearest neighbour were roughly similar, $b\left(x=0\right)\approx b\left(x=1\right)$, figure 4*e*, where $I_{A}\left(t=0.5\right)<I_{B}\left(t=0.5\right)$ despite $r_{A}>r_{B}$, however $I_{A}\left(t=1/12\right)>I_{B}\left(t=1/12\right)$ as inferred from $r_{A}>r_{B}$.

To explore the role of the size of the local area used in equation (2.7), we estimated r using areas of two different sizes and compared the quantities, exp⁡(r×Δt), Δt=1/12 year, with the mean number of infected fields I(Δt) obtained from simulations to determine the local area where the value of exp⁡(r×Δt) is closer to I(Δt). Here, the quantity exp⁡(r×Δt) provides the number of infected fields under the assumption of exponential growth with rate r, see insets in figures 4 and 5. We found that an estimate exp⁡(r×Δt) where r was calculated on the local area of a smaller size (i.e. 8 km-by-8 km) provided better agreement between estimates from computer simulations, I(Δt) and exp⁡(r×Δt), derived analytically from equation (2.7). Indeed, because all dispersal kernels, b(x), considered here decay significantly for x>2 km, the value $I\left(\Delta t\right)$ with Δt=1/12 year is strongly influenced by the area in close proximity (<8 km) to the initial infected field. This influence is captured better by estimations of r from equation (2.7) on the smaller area than on the larger area, because the latter is influenced by spatial heterogeneities that are far from the initially infected field and effectively do not contribute to the value $I\left(\Delta t\right)$ estimated from computer simulations. The exceptions were observed in cases where the pathogen dispersal is short-ranged (figures 4*e* and 5*c*–e) and therefore the assumption of long-ranged dispersal required by equation (2.7) is violated and the epidemic dynamics are far from exponential growth (hence the several orders of magnitude deviation between $I\left(\Delta t\right)$ and exp⁡(r×Δt) in figures 4*e* and 5*c*–e). Nevertheless, the relative ranking of $I\left(\Delta t\right)$ is captured correctly by estimates of r in figures 4*e* and 5*c*–e.

Overall, these results (from Model 2) show that the analytical estimates of the infection rate can be used for qualitative predictions (i.e. the relative ranking) of the effect of spatial structure of the landscape on an epidemic despite a complicated relationship between the host landscape and the pathogen dispersal kernel that previously could be captured only by computer simulations.

### 3.4. Identifying spatial configurations that decelerate invading pathogens

Here, we apply the analytical approach, using a hypothetical example to address a current and important challenge faced by food producers: Where should crops be deployed in a landscape to minimize risks from invading pathogens? Assuming that only a small fraction of the landscape (considered in the previous section) can be reconfigured by redistributing where susceptible crops are planted, we want to find the top-priority local areas for spatial reconfiguration of the crop that would provide the strongest deceleration of the invading pathogen. We formulate the problem in the following way. Consider the original cassava landscape, H (figure 3*a,*b), on two overlaying square lattices with mesh size 24 and 2 km, respectively, so that each 24 km-by-24 km cell contains 144 of the 2 km-by-2 km cells. Assume, that only five 2 km-by-2 km cells within each 24 km-by-24 km cell can undergo type B reconfiguration. We consider the problem of finding five 2 km-by-2 km cells within each 24 km-by-24 km cell where type B reconfiguration provides the strongest contribution towards the overall deceleration of the spread of CBSV.

We solve this problem by using analytical estimates of local infection rates introduced above. To calculate local infection rates, we consider the host landscape on a square lattice with mesh size 8 km, i.e. the size of a smaller area used for estimations of the infection rates in the previous section. We calculate the infection rate (equation 2.7) in each 8 km-by-8 km cell on the original landscape H and on the landscape B and obtain values $r_{H}$ and $r_{B}$, respectively. The value of the difference $\left(r_{B}-r_{H}\right)$ is used as a measure of the impact of spatial reconfiguration of the host landscape on the spread of the pathogen. Then, to quantify the contribution from a given 2 km-by-2 km area, the value of $\left(r_{B}-r_{H}\right)$ in the relevant 8 km-by-8 km cell is multiplied by the number of hosts in that 2 km-by-2 km area; this number of hosts is denoted by $N_{2km}$. The resulting quantity, $\left(r_{B}-r_{H}\right)\times N_{2km}$, is assigned to the appropriate 2 km-by-2 km cell and is proportional to its contribution to the overall impact of the spatial reconfiguration on the epidemic. The five cells with the largest values of $\left(r_{B}-r_{H}\right)\times N_{2km}$ represent the solution to the problem.

Note, if the infection rate is estimated on the whole 24 km-by-24 km area, then the solution to the problem is given simply by those five 2 km-by-2 km cells that have the highest host density, $N_{2km}$. This is because the value of $\left(r_{B}-r_{H}\right)$ in that case is the same for all 2 km-by-2 km cells within the 24 km-by-24 km area. By considering a smaller area for the estimation of the infection rate, the estimate becomes more accurate (as shown above), and the solution of the problem depends not only on the local host density but also on the spatial structure of the local neighbourhood. Therefore, in the solution presented here, only some of the selected five cells in 24 km-by-24 km area are cells with the highest host density.

**Figure 6.**
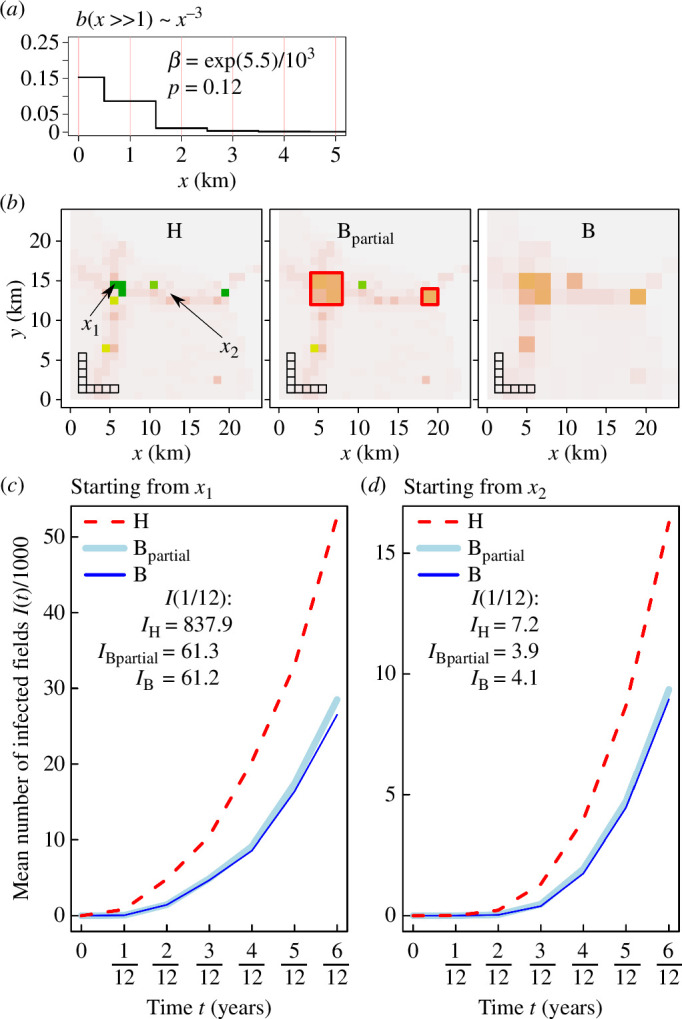
Illustration of use of analytical approximation to inform local reconfiguring of host landscape to reduce epidemic spread. (*a*) Pathogen dispersal kernel that corresponds to figure 4*c*. (*b*) Assuming that only a small fraction (approximately 3.5%, see text) of the host landscape can be reconfigured using type B, homogenization, we identified the preferred locations for local homogenization and constructed the corresponding landscape, denoted as B_partial_. The same sample area from three landscapes: the original (unmodified) landscape H, the partially homogenized landscape B_partial_ where the locally homogenized areas are outlined by the red boundary and the fully homogenized landscape B. Two different initial locations of a primary infection are denoted as $x_{1}$ and $x_{2}$ and shown in landscape H. (*c,d*) Results of computer simulations of Model 2 using the dispersal kernel (*a*), the three landscapes (H, B_partial_ and B) and two different primary locations of infection, $x_{1}$ and $x_{2}$. Here, the effect from type B reconfiguration is similar to the effect from increasing number of clusters $N_{clusters}$ in figure 1: in both cases, the infection rates are reduced. Results for landscapes B_partial_ and B appear nearly indistinguishable in both cases.

To verify the solution, we perform the type B reconfiguration in the top priority cells and obtain a partially reconfigured landscape denoted as ‘B partial’. We use computer simulations of the IBM of pathogen spread (Model 2) using the same dispersal kernel b(x) as in figure 4*c*, also reproduced in figure 6*a*. We compare epidemics in three landscapes: (i) the original landscape H; (ii) the landscape B; and (iii) the landscape ‘B partial’. Two locations for the primary infection are considered: $x_{1}$ (the same as in figure 4) and $x_{2}$ (the same as in electronic supplementary material, figures S7 and S8, supplementary note S7) shown in figure 6*b*; the results of computer simulations are shown in figure 6*c*,*d*, respectively corresponding to the two initial conditions.

Our results demonstrate that the size of an epidemic in the partially reconfigured landscape is very close to the size of an epidemic in the fully reconfigured landscape, thus confirming that the reconfiguration of a subset of key cells provides the dominant contribution towards deceleration of the invading pathogen. We conclude that, in principle, the method based on infection rate can identify landscape configurations that decelerate the spread of invading pathogens.

## 4. Discussion

Using the mathematical framework for the analysis of IBMs [30], we have derived an analytical approximation to infer the effect of the local spatial structure of a host landscape on epidemic dynamics. A major advantage of the analytical approximation over computer simulations is that it can be used to compare many different landscape reconfigurations quickly and to identify those that provide the strongest deceleration to the invasion of a given pathogen. To demonstrate this, we have compared the effect from two types of small-scale spatial reconfigurations of a host landscape on epidemic dynamics, using CBSV as an exemplar. Our results show that local scale spatial homogenization (Type B configuration, figure 3*b*) decelerates the epidemic. The result held for a range of plausible kernels (figures 3 and 4) derived from a posterior distribution reported for landscape scale spread of CBSV by Godding *et al.* [24]. Small-scale spatial aggregation (Type A configuration in figure 3*b*) decelerates the epidemic for some dispersal kernels (as in figure 4*e*) but accelerates it for other kernels (e.g. as in figure 5*e*). These results demonstrate: (i) the critical importance of the dispersal kernel relative to the spatial structure of the host landscape on epidemic dynamics; (ii) a complicated dependence of epidemic dynamics on the interaction between dispersal kernel and spatial structure of host landscapes; and (iii) that these effects on epidemic dynamics can be inferred by the analytical approximation presented in this article. Additionally, assuming limited resources are available for landscape reconfiguration, we have identified the preferred locations where type B local reconfiguration of fields should occur to decelerate epidemic spread (figure 6). We have shown that the partially reconfigured landscape decelerates the epidemic almost as effectively as for a landscape where local homogenization was applied everywhere (figure 6*c*,*d*).

The analytical approximation (equation 2.7) for the infection rate at the start of the epidemic relies on the assumption of sufficiently long-ranged dispersal of pathogens relative to the spatial structure of a host landscape. This assumption is valid for many plant pathogens whose dispersal has a strong long-distance component. The success of the approximation (equation 2.7) depends also on the discretization of the host landscape. Within Model 1 (for artificial landscapes) we have shown that dividing a crop landscape into coarser, individual host units for IBM reduced the accuracy of agreement between the analytical approximation and estimates from computer simulations (cf. figure 2*c*,*d*). Similarly, in Model 2 (for realistic landscapes) it is likely that the coarser the spatial resolution in a rasterized host landscape, the poorer the agreement would be. The spatial resolution of the rasterized data available for the current investigation [36] precluded analysis at finer spatial scales.

In addition, in heterogeneous landscapes, agreement between the analytical approximation (equation 2.7) and estimates from computer simulations depends on the size of the area in which r is calculated. We have shown that when the size of the area for calculating r is much larger that the scale of dispersal the deviations between estimates from equation (2.7) and computer simulations can be substantial. Hence, in heterogeneous landscapes r should be estimated locally on the area whose size depends on the spatial scale of dispersal. This observation is in line with earlier studies [25,31] where the local area has been used to estimate infection rate at the start of an epidemic: Bolker [25] introduced the local area as the inverse of the integral of the dispersal kernel squared; Suprunenko *et al.* [31] proposed to determine the area as a disc that would encompass 95% of the dispersal kernel. In this work, we estimated r on the same fixed size area regardless of a dispersal kernel. Further investigations are needed to fully understand the optimal selection of the local area for accurate estimation of infection rate analytically.

In earlier studies of the infection rate for epidemics [20,25,33], it was implicitly assumed that when introducing infection to a number S(t=0) of initially susceptible hosts the number I(0) is negligibly small compared with the total number, N, of all hosts. Therefore, in the derivation of the infection rate the number of susceptible hosts S(t=0), where S(0)=N-I(0), was effectively substituted by the total number of hosts, S(0)≈N. This could be related to the typical association of $R_{0}$ (and, consequently, r) with the leading eigenvalue of the corresponding equations linearized around disease-free equilibrium, i.e. linearized by assuming $I\left(t\right)\to 0$ as t→0 [16,17,20]. In this study, we took a different approach based on the idea of an invasion as a localized phenomenon [31]: when r (and, consequently, $R_{0}$) is estimated locally, the number of initially infected hosts I(0) within the local area may be a non-negligible fraction of the number N of all hosts within that area. Therefore, the distinction between S(0) and N in the approximation of the cumulant, $g_{SI}(x)$, as in equation (2.6), provides a more accurate approximation of the actual $g_{SI}(x)$ when the number N of hosts within the local area is sufficiently small (§2).

The results in this study are applicable also to the S(E)I(R)(S) family of epidemiological models (§2); in addition, the model could in principle be extended to include dynamical landscapes and crop rotation, e.g. using an approach of North & Godfray [20] in which individual hosts are created and removed stochastically with certain rates. We consider that the analytical approximation, equation (2.7), could be used to review the design of the spatial configuration of agricultural landscapes to increase resilience to emerging epidemics. In addition, the analytical approximation could in principle be used to inform and design the spatial deployment of disease control strategies [14,21,40], such deployment renders otherwise susceptible fields unavailable to the pathogen. This could be achieved by deploying resistant varieties [13] before the invasion of pathogens or spraying pesticides or fungicides at the early stages of the invasion.

## Data Availability

Data: [34]. Code: [35]. Supplementary material is available online [41].

## References

[1] Meyfroidt P *et al*. 2022 Ten facts about land systems for sustainability. Proc. Natl Acad. Sci. USA **119**, e2109217118. (10.1073/pnas.2109217118) 35131937 PMC8851509

[2] Phalan B, Onial M, Balmford A, Green RE. 2011 Reconciling food production and biodiversity conservation: land sharing and land sparing compared. Science **333**, 1289–1291. (10.1126/science.1208742) 21885781

[3] Grass I *et al*. 2019 Land‐sharing/‐sparing connectivity landscapes for ecosystem services and biodiversity conservation. People Nat. **1**, 262–272. (10.1002/pan3.21)

[4] Segre H, Carmel Y, Shwartz A. 2022 Economic and not ecological variables shape the sparing–sharing trade‐off in a mixed cropping landscape. J. Appl. Ecol. **59**, 779–790. (10.1111/1365-2664.14092)

[5] Correa Ayram CA, Mendoza ME, Etter A, Salicrup DRP. 2016 Habitat connectivity in biodiversity conservation. Prog. Phys. Geogr. **40**, 7–37. (10.1177/0309133315598713)

[6] Brennan A, Naidoo R, Greenstreet L, Mehrabi Z, Ramankutty N, Kremen C. 2022 Functional connectivity of the world’s protected areas. Science **376**, 1101–1104. (10.1126/science.abl8974) 35653461

[7] Cunniffe NJ, Cobb RC, Meentemeyer RK, Rizzo DM, Gilligan CA. 2016 Modeling when, where, and how to manage a forest epidemic, motivated by sudden oak death in California. Proc. Natl Acad. Sci. USA **113**, 5640–5645. (10.1073/pnas.1602153113) 27140631 PMC4878485

[8] Nguyen VA, Bartels DW, Gilligan CA. 2023 Modelling the spread and mitigation of an emerging vector-borne pathogen: citrus greening in the US. PLoS Comput. Biol. **19**, e1010156. (10.1371/journal.pcbi.1010156) 37267376 PMC10266658

[9] Savary S, Willocquet L, Pethybridge SJ, Esker P, McRoberts N, Nelson A. 2019 The global burden of pathogens and pests on major food crops. Nat. Ecol. Evol. **3**, 430–439. (10.1038/s41559-018-0793-y) 30718852

[10] Fones HN, Bebber DP, Chaloner TM, Kay WT, Steinberg G, Gurr SJ. 2020 Threats to global food security from emerging fungal and oomycete crop pathogens. Nat. Food. **1**, 332–342. (10.1038/s43016-020-0075-0) 37128085

[11] Jones RAC. 2021 Global plant virus disease pandemics and epidemics. Plants **10**, 233. (10.3390/plants10020233) 33504044 PMC7911862

[12] Ristaino JB *et al*. 2021 The persistent threat of emerging plant disease pandemics to global food security. Proc. Natl Acad. Sci. USA **118**, e2022239118. (10.1073/pnas.2022239118) 34021073 PMC8201941

[13] Rimbaud L, Fabre F, Papaïx J, Moury B, Lannou C, Barrett LG, Thrall PH. 2021 Models of plant resistance deployment. Annu. Rev. Phytopathol. **59**, 125–152. (10.1146/annurev-phyto-020620-122134) 33929880

[14] Gilligan CA, Truscott JE, Stacey AJ. 2007 Impact of scale on the effectiveness of disease control strategies for epidemics with cryptic infection in a dynamical landscape: an example for a crop disease. J. R. Soc. Interface **4**, 925–934. (10.1098/rsif.2007.1019) 17609179 PMC1975768

[15] Mundt CC, Sackett KE, Wallace LD. 2011 Landscape heterogeneity and disease spread: experimental approaches with a plant pathogen. Ecol. Appl. **21**, 321–328. (10.1890/10-1004.1) 21563564

[16] Mikaberidze A, Mundt CC, Bonhoeffer S. 2016 Invasiveness of plant pathogens depends on the spatial scale of host distribution. Ecol. Appl. **26**, 1238–1248. (10.1890/15-0807) 27509761

[17] Shigesada N, Kawasaki K, Teramoto E. 1986 Traveling periodic waves in heterogeneous environments. Theor. Popul. Biol. **30**, 143–160. (10.1016/0040-5809(86)90029-8)

[18] Shigesada N, Kawasaki K, Teramoto E. 1987 The speeds of traveling frontal waves in heterogeneous environments. In Lecture notes in biomathematics mathematical topics in population biology, morphogenesis and neurosciences, pp. 88–97. Berlin Heidelberg: Springer. (10.1007/978-3-642-93360-8_9)

[19] Hastings A *et al*. 2005 The spatial spread of invasions: new developments in theory and evidence. Ecol. Lett. **8**, 91–101. (10.1111/j.1461-0248.2004.00687.x)

[20] North AR, Godfray HCJ. 2017 The dynamics of disease in a metapopulation: the role of dispersal range. J. Theor. Biol. **418**, 57–65. (10.1016/j.jtbi.2017.01.037) 28130098 PMC5360276

[21] Park AW, Gubbins S, Gilligan CA. 2001 Invasion and persistence of plant parasites in a spatially structured host population. Oikos **94**, 162–174. (10.1034/j.1600-0706.2001.10489.x)

[22] Keeling MJ. 1999 The effects of local spatial structure on epidemiological invasions. Proc. Biol. Sci. **266**, 859–867. (10.1098/rspb.1999.0716) 10343409 PMC1689913

[23] Neuhauser C. 2001 Mathematical challenges in spatial ecology. Notices. AMS. **48**, 1304–1314. https://www.ams.org/notices/200111/fea-neuhauser.pdf

[24] Godding D, Stutt R, Alicai T, Abidrabo P, Okao-Okuja G, Gilligan CA. 2023 Developing a predictive model for an emerging epidemic on cassava in sub-saharan Africa. Sci. Rep. **13**, 12603. (10.1038/s41598-023-38819-x) 37537204 PMC10400665

[25] Bolker BM. 1999 Analytic models for the patchy spread of plant disease. Bull. Math. Biol. **61**, 849–874. (10.1006/bulm.1999.0115) 17886747

[26] Brown DH, Bolker BM. 2004 The effects of disease dispersal and host clustering on the epidemic threshold in plants. Bull. Math. Biol. **66**, 341–371. (10.1016/j.bulm.2003.08.006) 14871569

[27] Plank MJ, Law R. 2015 Spatial point processes and moment dynamics in the life sciences: a parsimonious derivation and some extensions. Bull. Math. Biol. **77**, 586–613. (10.1007/s11538-014-0018-8) 25216969

[28] Ovaskainen O, Cornell SJ. 2006 Space and stochasticity in population dynamics. Proc. Natl Acad. Sci. USA **103**, 12781–12786. (10.1073/pnas.0603994103) 16912114 PMC1568924

[29] Ovaskainen O, Finkelshtein D, Kutoviy O, Cornell S, Bolker B, Kondratiev Y. 2014 A general mathematical framework for the analysis of spatiotemporal point processes. Theor. Ecol. **7**, 101–113. (10.1007/s12080-013-0202-8)

[30] Cornell SJ, Suprunenko YF, Finkelshtein D, Somervuo P, Ovaskainen O. 2019 A unified framework for analysis of individual-based models in ecology and beyond. Nat. Commun. **10**, 4716. (10.1038/s41467-019-12172-y) 31624268 PMC6797757

[31] Suprunenko YF, Cornell SJ, Gilligan CA. 2021 Analytical approximation for invasion and endemic thresholds, and the optimal control of epidemics in spatially explicit individual-based models. J. R. Soc. Interface **18**, 20200966. (10.1098/rsif.2020.0966) 33784882 PMC8086857

[32] Wadkin LE, Holden J, Ettelaie R, Holmes MJ, Smith J, Golightly A *et al*. 2024 Estimating the reproduction number, R 0, from individual-based models of tree disease spread. Ecol. Modell. **489**, 110630. (10.1016/j.ecolmodel.2024.110630)

[33] Bosch F, Helps J, Cunniffe NJ. 2024 The basic‐reproduction number of infectious diseases in spatially structured host populations. Oikos **2024**, e10616. https://nsojournals.onlinelibrary.wiley.com/doi/10.1111/oik.10616

[34] Suprunenko YF, Cornell SJ, Gilligan CA. 2024 Data from: predicting the effect of landscape structure on epidemic invasion using an analytical estimate for infection rate. Figshare. (10.6084/m9.figshare.25804702) PMC11706665 39780974

[35] Suprunenko YF, Cornell SJ, Gilligan CA. 2024 Computer code for: predicting the effect of landscape structure on epidemic invasion using an analytical estimate for infection rate. Figshare (10.6084/m9.figshare.25804810) PMC11706665 39780974

[36] Szyniszewska AM. 2020 CassavaMap, a fine-resolution disaggregation of cassava production and harvested area in Africa in 2014. Sci. Data **7**, 159. (10.1038/s41597-020-0501-z) 32461559 PMC7253458

[37] Nweke FI, Lynam JK, Spencer DSC. 2002 The cassava transformation: Africa’s best kept secret. Michigan State University Press. See https://www.jstor.org/stable/10.14321/j.ctt7ztc0t.

[38] Alicai T, Omongo CA, Maruthi MN, Hillocks RJ, Baguma Y, Kawuki R, Bua A, Otim-Nape GW, Colvin J. 2007 Re-emergence of cassava brown streak disease in uganda. Plant Dis. 91, 24–29. (10.1094/pd-91-0024) 30781061

[39] Muhindo H, Wembonyama F, Yengele O, Songbo M, Tata-Hangy W, Sikirou M, Pita J, Monde G. Optimum Time for harvesting cassava tubers to reduce losses due to cassava brown streak disease in Northeastern DRC. J.A.S. **12**, 70. (10.5539/jas.v12n5p70)

[40] Jeger MJ, Madden LV, Bosch F. 2018 Plant virus epidemiology: applications and prospects for mathematical modeling and analysis to improve understanding and disease control. Plant Dis. **102**, 837–854. (10.1094/pdis-04-17-0612-fe) 30673389

[41] Suprunenko Y, Cornell S, Gilligan C. 2024 Supplementary material from: Predicting the effect of landscape structure on epidemic invasion using an analytical estimate for infection rate. Figshare. (10.6084/m9.figshare.c.7569508) PMC11706665 39780974

